# Conjugal Affection

**Published:** 1855-03

**Authors:** 


### CONJUGAL AFFECTION.

### A CONDITION OF THE SUCCESSFUL TRAINING OF CHILDREN.

Without strong conjugal affection on the part of parents, there cannot be that cordial co-operation in the education of their offspring—one of the most important of the trusts and duties of wedded life, which is essential to success. This want of cordial co-operation may not be distinctly *seen* by the children, but, what is scarcely better, it will be *felt* by them, and will produce an unhappy effect upon their dispositions. If there is, as sometimes happens, undisguised dissonance, this will be both seen and felt; and all the charms of home, all its genial influences will be wanting. For the children of such parents there is no true home, and there are no loving impulses pressing them toward the paths of virtue and of peace. And even when there is only a coldness and formality subsisting between the husband and the wife, this is sufficient to render the atmosphere of home too cold and frosty for the growth of childlike virtues. It cannot be too deeply realized, that a happy home in childhood is as needful for the growth of lovely and sweet dispositions, as the genial warmth of spring to the development of the budding beauty that then adorns the earth. But conjugal affection is the first element of family happiness. Without it, children may not know where the difficulty lies, but they will feel that the little world of home does not move on harmoniously; they will be deprived of that home felicity which is their rightful inheritance, and necessary to the development and growth of some of the best dispositions of the heart. Besides, as age advances, they will learn by degrees that their parents do not love each other; and, with this knowledge, there will be likely to come the partisan feeling which, instead of honoring both their father and their mother, will love the one and hate the other, or else cleave to the one and despise the other. Another most pernicious consequence is apt to follow from this want of affection. It interweaves with the earliest associations of children that doctrine of devils and of immortal ruin, that connubial happiness is the dream of poetry, or of youthful love, never to be realized in actual life —a dream from which those who dream it will wake, if they are ever married, and find it but a dream. Beautiful and important, in every point of view—whether we regard the happiness of the domestic circle, or the virtues which should there bud, blossom, and ripen into fruit—is the divine direction, "Husbands, love your wives, even as Christ loved His Church; and let the wife see that she reverence her husband:" for, if she do this, her woman's heart cannot fail to love him.—*Magazine for Mothers.*

